# Strength and Degradation Characteristics of Zein Biopolymer-Treated Sands Under Wetting–Drying Cycles

**DOI:** 10.3390/polym18070888

**Published:** 2026-04-05

**Authors:** Quadri Olakunle Babatunde, Woonjae Yeo, Yong-Hoon Byun

**Affiliations:** Department of Agricultural Civil Engineering, Kyungpook National University, 80 Daehak-Ro, Buk-Gu, Daegu 41566, Republic of Korea

**Keywords:** biopolymer, degradation, durability, compressive strength, wetting–drying cycle

## Abstract

Repeated wetting–drying cycles accelerate scouring and deteriorate soil structure by increasing pore-water pressure. This study examines the durability of sand treated with zein biopolymers subjected to wetting–drying cycles and compares its uncycled condition with that of xanthan gum (XG). The treated specimens are prepared with biopolymer contents of 1% and 3% by mass of sand. The specimens are cured for an initial period of 7 days under atmospheric conditions, whereafter they are subjected to a series of wetting–drying cycles. Subsequently, the dimensions and mass of the specimens are measured to evaluate bulk density-related changes during the cycles. The strength and degradation characteristics of the specimens are evaluated through unconfined compression tests after being subjected to different numbers of cycles. The bulk unit weight after the drying phase remains nearly constant, whereas that after the wetting phase increases with both the number of cycles and biopolymer content. Overall, specimens with higher biopolymer content exhibit lower bulk unit weights. The XG-treated specimens show earlier strength improvement than the zein-treated specimens due to the faster curing-related strength development associated with water-based gelation. Moreover, the XG-treated sand rapidly fails after the first wetting phase, while the compressive strength of the cycled zein-treated specimens is lower than that of the uncycled specimens. Zein-treated sand with 3% biopolymer content shows a higher durability index after 10 cycles than sand treated with 1% biopolymer content. Therefore, a higher zein content can be used to enhance the durability of sand subject to frequent wetting and drying cycles.

## 1. Introduction

The removal of soil particles under repeated wetting–drying cycles can degrade soil structure and increase susceptibility to scour and erosion [1,2]. In addition, extreme hydrological events can intensify wetting–drying actions and accelerate the detachment of soil particles by increasing pore water pressure [1,3,4]. Traditional stabilization methods, such as riprap, grout mats, and concrete aprons, have been introduced to mitigate scouring around hydraulic structures [5]. Although these traditional methods have been widely used to enhance the structural stability of soils, their application is costly and contributes significantly to carbon emissions [2,6]. In addition, synthetic polymer stabilizers have been used to improve the strength characteristics of soils. However, their use has raised environmental concerns due to potential toxicity and non-biodegradability [7]. Therefore, biopolymer-based stabilizers have emerged as sustainable alternatives due to their natural origin, non-toxicity, and ability to form strong gel networks [8,9].

Polysaccharide-based biopolymers, such as agar, guar gum, and xanthan gum, have been used to improve the compressive strength, erosion resistance, and stiffness of treated soils [9,10,11]. The bonding mechanism of these biopolymers involves the hydration of long-chain polysaccharide molecules, forming viscous hydrogels that coat soil particles and fill pore spaces within the soil matrix [12,13]. This gelation process results in rapid bonding, significantly increasing stiffness and resistance to mechanical loading [13,14]. However, the stiffness of polysaccharide-based hydrogels gradually decreases during wetting–drying cycles due to their hydrophilic nature. Their molecular structure is dominated by hydroxyl groups, which readily interact with water [7,15]. Several studies have reported that repeated wetting and drying can progressively weaken the hydrogel structure and reduce the mechanical performance of biopolymer-treated soils [16,17,18]. In addition, although polysaccharide-treated soils may retain some improvement, their susceptibility to shrink–swell stresses remains a major limitation under fluctuating moisture conditions [12,15,16]. Therefore, the degradation of polysaccharide-treated soils under cyclic moisture variations may limit their field application.

Protein-based biopolymers have recently attracted attention as soil stabilizers due to their hydrophobic gelation and enhanced strength development [19,20]. The protein-based biopolymers possess amphiphilic molecular structures dominated by hydrophobic amino acid groups, which can improve moisture resistance and bonding with sand particles [21,22]. The gelation mechanism of protein-based biopolymers involves protein denaturation after reacting with the solvent, resulting in pore filling by biopolymer gel within the sand matrix [19,23,24]. Protein-based biopolymers, such as casein, soy protein, and zein, have been used to improve the strength and stiffness characteristics of soils due to their stronger gel formation and hydrophobic characteristics compared to polysaccharide-based biopolymers [19,25,26,27]. Recently, zein has been used to enhance the erosion resistance, apparent cohesion, and friction angle of sand [28,29]. The strength improvement of zein-treated sand depends on the uniformity of the sand and the consistency index of the gel [19,28]. Nevertheless, the durability characteristics of zein-treated soil under repeated wetting–drying cycles require further investigation for potential application in infrastructure exposed to seasonal moisture fluctuations.

This study investigates the durability characteristics of sand treated with two biopolymer binders, xanthan gum and zein, under repeated wetting and drying cycles. First, the gradation and index properties of the silica sand, as well as the physical characteristics of the two biopolymers, are presented. Then, the specimen preparation procedure and the testing program for the wetting–drying cycles, volumetric measurements, and unconfined compression tests are described. Changes in bulk density, stress–strain behavior, and compressive strength are evaluated as functions of biopolymer type, biopolymer content, curing period, and number of cycles. Based on the results, the early-stage response of xanthan gum-treated sand is compared with that of zein-treated sand, while the degradation behavior and durability of zein-treated sand under repeated wetting–drying cycles are further quantified using ductility and durability indices.

## 2. Biopolymer-Treated Soil

### 2.1. Biopolymers

**Figure 1:**
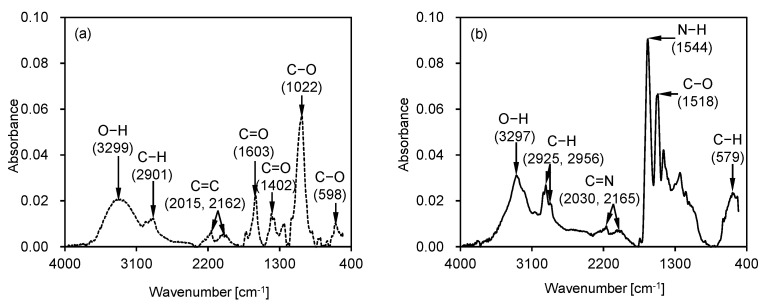
Fourier transform infrared spectroscopy of two biopolymers: (**a**) xanthan gum; (**b**) zein.

Biopolymers are macromolecules consisting of repeating long-chain monomers extracted from living organisms, such as plants, animals, and bacteria [19,21]. In this study, two types of biopolymers, a polysaccharide-based xanthan gum and a protein-based zein, were used. Table 1 summarizes the physical properties of these biopolymers, while Figure 1 presents their Fourier-transform infrared spectra.

Xanthan gum (XG) is an extracellular polysaccharide-based biopolymer synthesized by *Xanthomonas campestris* through aerobic fermentation of carbohydrates [32,33]. The extraction of xanthan gum involves fermentation, followed by alcohol precipitation and drying [31]. Xanthan gum’s molecular structure consists of a β-D-glucose backbone with trisaccharide side chains that contain glucuronic acid and mannose, which are frequently altered by acetyl and pyruvate groups [32]. As shown in Figure 1a, the presence of hydroxyl and carboxyl functional groups in xanthan gum contributes to its solubility and ionic interactions. Xanthan gum exhibits consistent non-Newtonian shear-thinning behavior, regardless of the curing conditions [32]. Moreover, its viscosity remains almost constant across a wide range of pH, temperature, and ionic conditions.

Zein is a hydrophobic prolamin protein extracted from corn endosperm using aqueous ethanol [19,21]. The gelation mechanism of zein involves reactions with polar solvents to form a three-dimensional protein network [29]. Its molecular structure consists of α-, β-, γ-, and δ-zein fractions [25]. As shown in Figure 1b, the presence of amide, hydroxyl, and carboxyl functional groups enhances the chemical interactions and crosslinking behavior of zein [34]. Zein gel demonstrates viscoelastic, non-Newtonian shear-thinning or shear-thickening behavior, depending on its solvent and biopolymer content [21]. The bonding between the biopolymer and soil occurs mainly through particle coating and pore filling by the viscous gel, regardless of the molecular structure. Consequently, the bonding strength of biopolymer binders significantly depends on several factors, such as molecular weight, viscosity, and curing conditions [16,25].

### 2.2. Soil

**Figure 2:**
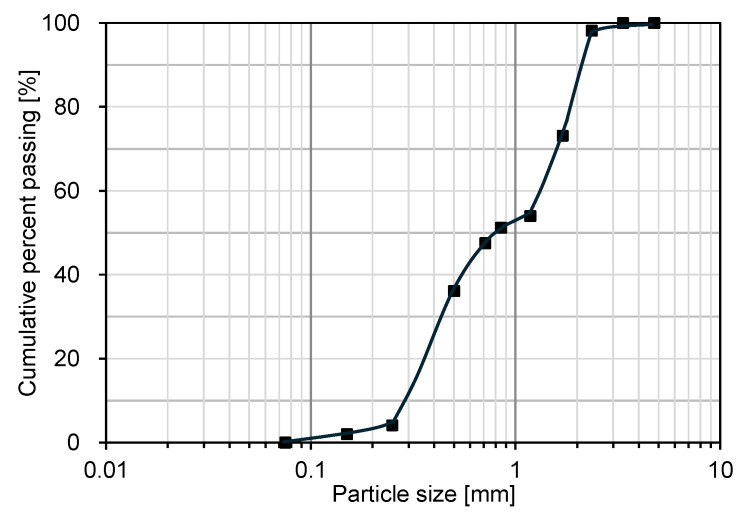
Grain size distribution curve of the sand used in this study.

Cohesionless silica sand was used to evaluate the durability of the biopolymer treatment due to its highly permeable structure. The grain-size distribution curve of the sand, obtained from sieve analysis conducted in accordance with ASTM D6913 [35], is shown in Figure 2, with a mean diameter of 0.80 mm. The smooth curve is intended as a visual guide to represent the overall gradation trend, rather than a fitted analytical model. According to the Unified Soil Classification System, the sand is classified as poorly graded (SP). Based on the compaction properties of the untreated sand, determined through a standard Proctor test in accordance with ASTM D698 [36], its maximum dry unit weight was 19.1 kN/m^3^.

### 2.3. Specimen Preparation

The sand specimens were prepared with two biopolymer contents, 1% and 3% by mass of dry sand. The dry sand was first mixed with the biopolymer powder using an electric mixer to ensure homogeneity. Subsequently, 9.5% solvent by mass was added to initiate biopolymer dissolution. The two biopolymers, xanthan gum and zein, were prepared with two solvents, water and ethanol–water, both in the same volume, selected based on their respective solubility characteristics. The moist specimens were compacted into cylindrical molds (100 mm in height and 50 mm in diameter) in three equal layers. Table 2 summarizes the dry unit weights of the biopolymer-treated sands using the procedure provided by ASTM D698 [36]. The specimens were initially cured under atmospheric conditions at 23 ± 2 °C and 40% ± 5% relative humidity. The volumetric changes and strength characteristics of the cylindrical specimens were subsequently evaluated throughout the curing period and wetting–drying cycles.

## 3. Experimental Study

**Figure 3:**
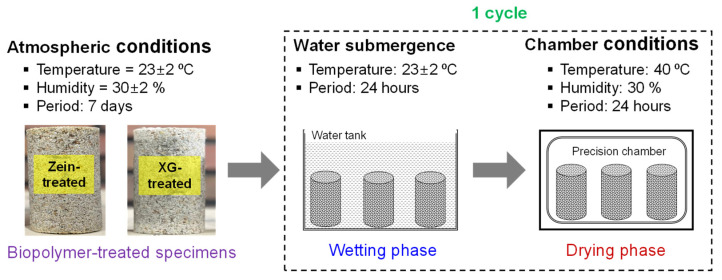
Schematic diagram of the wetting–drying process.

**Figure 4:**
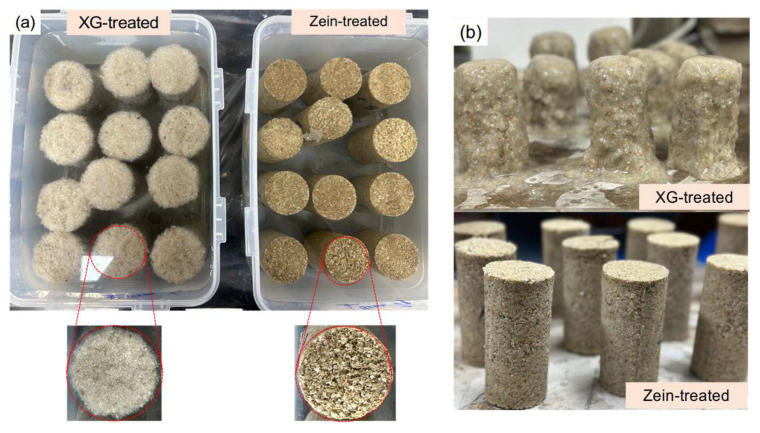
Biopolymer-treated specimens at different curing periods: (**a**) 12 h (under immersion); (**b**) 24 h (after immersion).

The variation in soil strength under wetting–drying cycles is essential for evaluating the durability of stabilized soils under moisture fluctuations. In this study, the specimens were subjected to wetting–drying cycles after 7 days of curing, in accordance with ASTM D559 [37]. Each cycle consisted of a wetting phase and a drying phase, with a total duration of 48 h, as shown in Figure 3. During the wetting phase, the specimens were submerged in a water bath at 23 ± 2 °C for 24 h. A total of 12 specimens were fully immersed in a 10 L water bath to maintain a water-to-specimen volume ratio of 4:1, as shown in Figure 4. This excess water volume ensured complete submergence and uniform saturation of all treated specimens throughout the wetting phase. The dimensions and mass of each specimen were measured 60 s after removal from the water bath, allowing excess surface moisture to drain. Thereafter, the wet specimens were dried in a controlled chamber at 40 °C and 30% relative humidity for another 24 h. This procedure is similar to that specified in ASTM D559 [37] and the method used by Chang et al. (2018), in which specimens are fully saturated using an excess volume of water during durability testing. In addition, the drying temperature of 40 °C was selected, which is slightly lower than the ASTM D559 [37] recommended temperature of 71 °C, in order to evaluate the durability characteristics of zein biopolymer-treated sand under milder drying conditions. Three wetting–drying conditions (3, 7, and 10 cycles) were used to simulate environmental moisture fluctuations. The bulk density changes and strength characteristics of the specimens after the wetting–drying cycles were evaluated through dimensional and mass measurements and unconfined compression testing, respectively. Note that the XG-treated specimens failed after 24 h, as shown in Figure 4b. Therefore, the durability characteristics of only the zein-treated sand were evaluated under wetting–drying conditions.

The bulk density of the specimens was evaluated under repeated wetting–drying cycles. After demolding, the dimensions and mass of each specimen were measured using a digital caliper with a resolution of 0.01 mm and a precision balance, respectively. These repeated measurements were used to monitor density changes associated with moisture absorption and particle detachment, as suggested in previous studies [18,33]. As the specimens were not oven-dried to a fully dried state during the cycling process, the measured values should be interpreted as bulk density changes based on the recorded mass and dimensions at each stage. These variations were used to analyze the density-related behavior of each specimen during the wetting–drying cycles.

The strength characteristics and durability indices of the treated specimens were evaluated using unconfined compression tests in accordance with ASTM D2166 [38]. The treated specimens were loaded axially at a constant displacement rate of 1 mm/min until failure. The axial load and displacement were recorded during the testing. The compressive strength was calculated as the maximum axial load divided by the cross-sectional area of the treated specimen. Three replicate specimens were tested under each condition to ensure reliable and reproducible results. The strength characteristics and durability indices of the specimens were determined as the average of replicate specimens. The durability index was calculated as the percentage ratio of the compressive strength of the cycled specimens to that of the uncycled specimens for the same curing period. Note that the uncycled specimens were those cured under atmospheric conditions, whereas the cycled specimens were those subjected to repeated wetting–drying cycles.

## 4. Results and Analysis

### 4.1. Density Change

**Figure 5:**
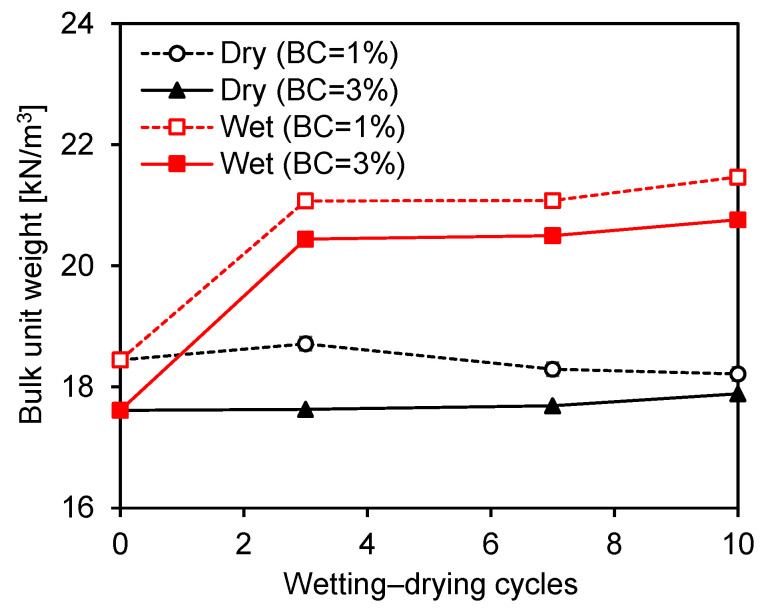
Variation in bulk unit weights of zein-treated specimens with the number of wetting–drying cycles. Dry and Wet denote the drying and wetting phases, respectively.

The changes in the bulk unit weights of the zein-treated specimens were monitored at the end of the wetting and drying phases of each cycle. The variations in bulk unit weight under repeated wetting–drying cycles are presented in Figure 5. The zein-treated specimens with higher biopolymer contents showed lower unit weights due to an increase in the biopolymer-to-soil ratio. Overall, the bulk unit weight measured after the drying phase remained nearly constant throughout the wetting–drying cycles, regardless of the biopolymer content. In contrast, the bulk unit weight measured after the wetting phase increased substantially with both the number of wetting–drying cycles and the biopolymer content. During the wetting phase, detachment of unbonded fine particles altered the pore structure and facilitated additional water penetration, which led to an increase in the measured bulk unit weight. Nevertheless, the gelation of the zein biopolymer produced strong interparticle bonds that mitigated excessive pore expansion and particle loss under repeated wetting–drying cycles.

### 4.2. Stress–Strain Behavior

**Figure 6:**
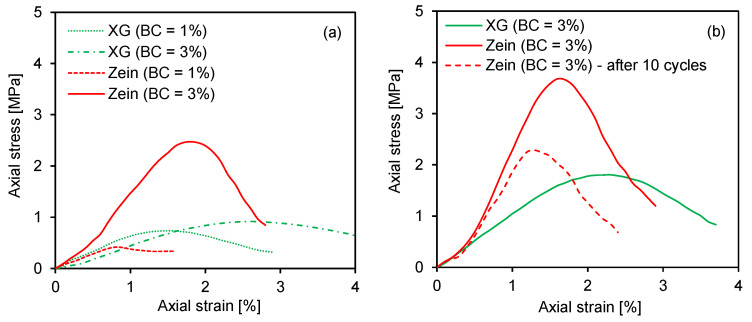
Stress–strain relationships of biopolymer-treated specimens at two curing periods: (**a**) 7 days; (**b**) 28 days.

The stress–strain behavior of the treated specimens is shown in Figure 6. The axial stress increased with strain until it reached a peak, corresponding to the compressive strength, after which it gradually decreased. During the initial curing period of 7 days, both the maximum axial stress and the corresponding strain increased with the biopolymer content, as shown in Figure 6a. For the 3% biopolymer content, the zein-treated specimen exhibited a higher peak stress and lower strain than the XG-treated specimen. After 28 days of curing, the maximum axial stress increased compared with that after 7 days of curing. However, after 10 wetting–drying cycles, the maximum axial stress and the corresponding strain of the zein-treated specimens slightly decreased, as shown in Figure 6b. The reduction in axial strain can be ascribed to the weakening of interparticle bonding induced by cyclic moisture variations. These findings align with Chen et al.’s [16] observation that cyclic moisture variations degrade the biopolymer–soil network and reduce interparticle cohesion. Notably, the peak axial stress of the zein-treated sand subjected to wetting–drying was higher than that of the uncycled XG-treated sand.

### 4.3. Strength Development

**Figure 7:**
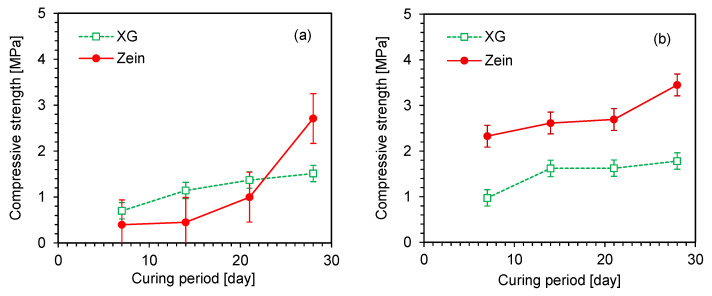
Unconfined compressive strength of uncycled biopolymer-treated specimens with two biopolymer contents: (**a**) BC = 1%; (**b**) BC = 3%.

The change in the compressive strength of the uncycled specimens with curing period is shown in Figure 7. The compressive strength of all specimens increased with both the curing period and biopolymer content. The strength improvement with increasing biopolymer content was more pronounced in the zein-treated specimens than in the XG-treated specimens, for which the difference between 1% and 3% biopolymer contents was marginal. At 1% biopolymer content, the XG-treated specimens exhibited rapid strength gain during the initial 7 days, followed by a gradual increase up to 28 days, whereas the zein-treated specimens showed a marked increase in strength after 28 days of curing. The delayed strength development of the zein-treated specimens is related to the solvent characteristics and the molecular structure of the zein binder. In the XG-treated specimens, the water solvent rapidly evaporated during curing, forming a viscous hydrogel, and the remaining water gradually dissipated, resulting in only a minor strength improvement beyond 7 days. Conversely, the zein-treated sand exhibited delayed strength development due to the protein-based molecular structure of zein, which requires a longer period for aggregation and transition into a rigid gel [39]. During early curing, the zein gel behaved as a viscous material owing to incomplete molecular rearrangement. Moreover, ethanol diffused slowly through the pore spaces due to its larger molecular size and weaker affinity for soil than water [40]. As a result, residual ethanol persisted for a longer period in the zein-treated specimens, thereby retarding soil-biopolymer bonding. Similarly, Babatunde et al. [25] reported lower compressive strength under atmospheric curing conditions because of residual moisture, as observed in scanning electron microscopy images. In addition, ethanol diffusion under gel conditions is gradual because of slow molecular rearrangement [41]. The hydrophobic zein network also reduces liquid permeability, thereby delaying the removal of remaining pore water from the zein-treated sand. This led to slower overall drying and delayed strength development compared with the XG-treated sand. For the biopolymer contents of both 1% and 3%, the zein-treated sand exhibited higher compressive strength after 28 days of curing than the XG-treated sand.

**Figure 8:**
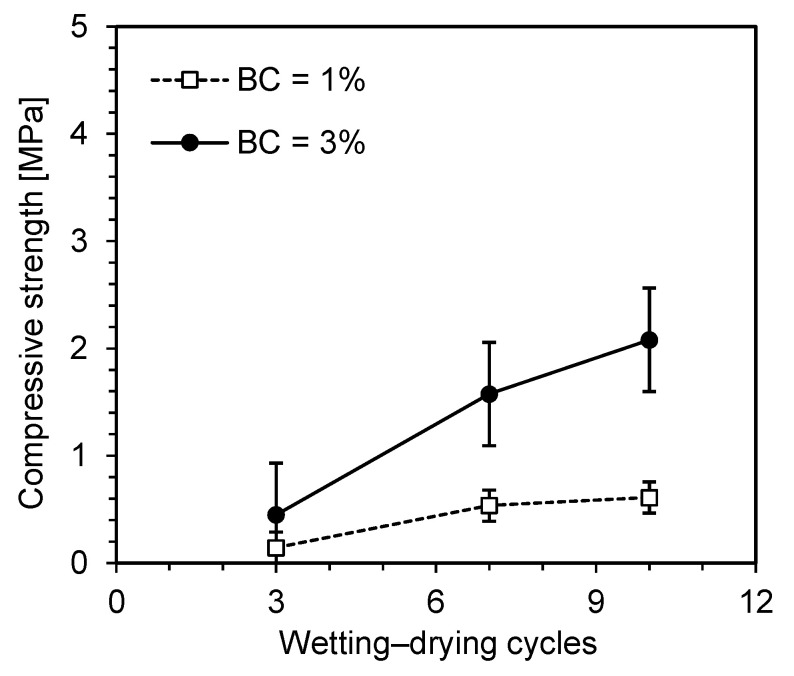
Unconfined compressive strength of zein-treated specimens after wetting–drying cycles.

The compressive strength of the zein-treated specimens subjected to repeated wetting–drying cycles is presented in Figure 8. The XG-treated specimens failed after the first wetting phase due to their strong affinity for water, as shown in Figure 4b. Therefore, only the zein-treated specimens were compared under cycled and uncycled conditions. The compressive strength of the cycled specimens increased with the number of wetting–drying cycles. In general, water penetration into soil pores weakens soil–biopolymer bonding and delays zein aggregation. However, solvent evaporation during the drying phase enhanced zein aggregation and strengthened intermolecular bonding. Nevertheless, repeated wetting–drying cycles retarded the gelation and aggregation within the soil matrix. These observations are consistent with those of Zou et al. [42], who reported that increased water content in zein gel disrupts hydrophobic interactions among protein molecules, thereby delaying gelation and network formation. Similarly, Chatsisvili et al. [43] found that high water content reduces the mobility of hydrophobic regions, hindering the rapid formation of rigid, glassy gels.

**Figure 9:**
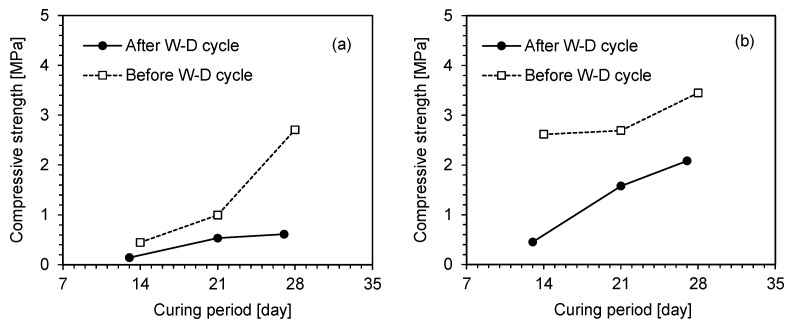
Variation in unconfined compressive strength of zein-treated specimens with two biopolymer contents: (**a**) BC = 1%; (**b**) BC = 3%. W-D cycle denotes the wetting–drying cycle.

A comparative analysis of the cycled and uncycled zein-treated specimens is presented in Figure 9. The compressive strength of all specimens increased with both the curing period and biopolymer content. The total curing periods corresponding to 3, 7, and 10 wetting–drying cycles are 13, 21, and 27 days, respectively. The compressive strength of the cycled specimens was lower than that of the uncycled specimens because of repeated wetting–drying. At 1% biopolymer content, the strength difference between the cycled and uncycled specimens was marginal during the initial cycles but became substantial after the 10th cycle. The reduction in compressive strength after multiple wetting–drying cycles can be attributed to delayed zein gelation caused by ethanol evaporation and the penetration of water molecules into the micropores. Hoy et al. [44] and Liu et al. [45] reported a similar reduction in the compressive strength of cement-stabilized soils due to water penetration, which weakens interparticle bonding. This finding is also consistent with the behavior of polyvinyl alcohol-stabilized soil reported by Li et al. [46]. Despite this reduction, the continued gelation of zein still contributed to strength development under repeated wetting–drying cycles. Compared with the specimens prepared with 1% biopolymer content, those prepared with 3% biopolymer content exhibited higher compressive strength under both cycled and uncycled conditions. Previously proposed polysaccharide-based biopolymer binders have been reported to fail rapidly under saturated conditions due to their affinity for water molecules [17,47]. Thus, the protein-based biopolymer binder zein used in this study demonstrated improved resistance to moisture fluctuations.

### 4.4. Degradation Characteristics

**Figure 10:**
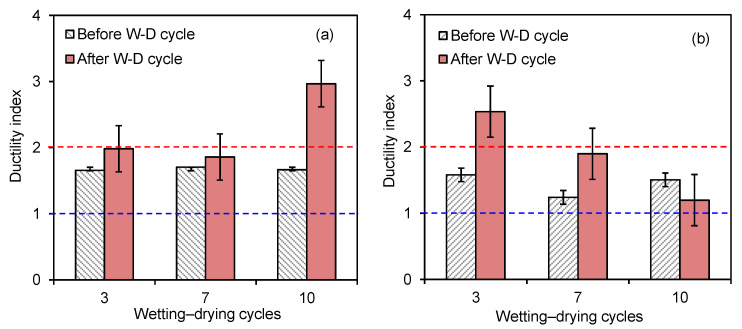
Ductility indices of zein-treated sands at two biopolymer contents: (**a**) BC = 1%; (**b**) BC = 3%. Blue and red dash-dotted lines denote brittleness and ductility boundary, respectively.

The variations in the ductility indices of the zein-treated specimens with two different biopolymer contents are presented in Figure 10. The ductility index represents the deformation behavior of treated specimens under axial loading [48,49]. It was determined as the ratio of the strain at 95% of the post-peak stress (*ε*_95_) to the strain at 50% of the peak compressive strength (*ε*_50_), in accordance with previous studies [50,51]. The empirical equation for the ductility index (*DCI*) can be expressed as follows:

(1) $$DCI=\frac{\varepsilon_{95}}{\varepsilon_{50}}\times 100\%\;$$

A ductility index lower than 1 indicates brittle behavior, whereas values greater than 2 indicate ductile behavior [52]. All zein-treated specimens exhibited moderately ductile behavior, which varied with both the biopolymer content and the number of wetting–drying cycles. Overall, the cycled specimens showed a higher ductility index than the uncycled specimens. After the third cycle, the specimens with 3% biopolymer content showed a higher ductility index than those with 1% biopolymer content. This behavior can be attributed to the relatively dense zein network formed around the soil particles, which enhanced interparticle bonding. The ductility index of the specimens generally increased with the number of cycles for the specimens with 1% biopolymer content, but decreased for those with 3% biopolymer content under moisture variations. For instance, the ductility index of the specimens with 3% biopolymer content decreased substantially, from 2.5 at the third cycle to 1.2 after the tenth cycle. At the higher biopolymer content, the specimens absorbed a limited amount of water due to the hydrophobicity and aggregation of zein, which increased their brittleness after drying. Chang et al. [10] also reported that excessive biopolymer content increases stiffness but reduces ductility under cyclic environmental conditions.

The durability index was used to evaluate the long-term stability of the treated specimens under fluctuating environmental conditions. Based on previous studies [53,54] the durability index (*DRI*) can be expressed as the ratio of the compressive strength of cycled specimens to that of uncycled specimens:

(2) $$\mathrm{DRI}=\frac{{\mathrm{UCS}}_{\mathrm{cycled}}}{{\mathrm{UCS}}_{\mathrm{uncycled}}}\times 100\%\;$$

**Figure 11:**
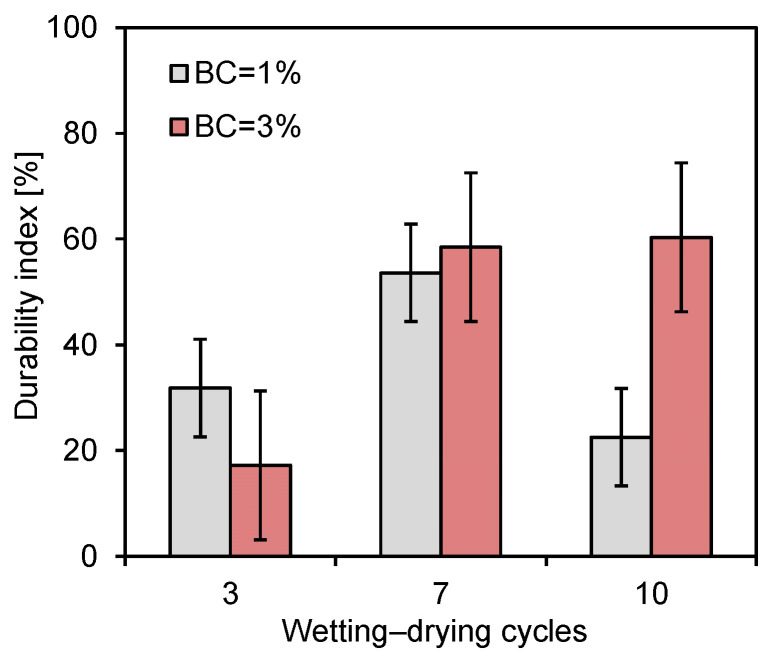
Durability indices of zein-treated sands under wetting–drying cycles.

The durability index of the treated specimens varied with the number of wetting–drying cycles and the biopolymer content, as shown in Figure 11. The specimen with 1% biopolymer content exhibited an inconsistent durability index with respect to the number of cycles. At the biopolymer content of 1%, the gelation between soil particles was non-uniform due to the inadequate biopolymer-to-soil ratio, which resulted in weak and discontinuous interparticle bonding. Consequently, repeated wetting–drying cycles caused particle detachment and reduced the durability index after the seventh cycle. In contrast, for the specimens with 3% biopolymer content, the durability index increased notably between the third and seventh cycles and remained nearly constant thereafter. This trend can be attributed to the progressive gelation of the zein biopolymer under wetting–drying cycles.

**Figure 12:**
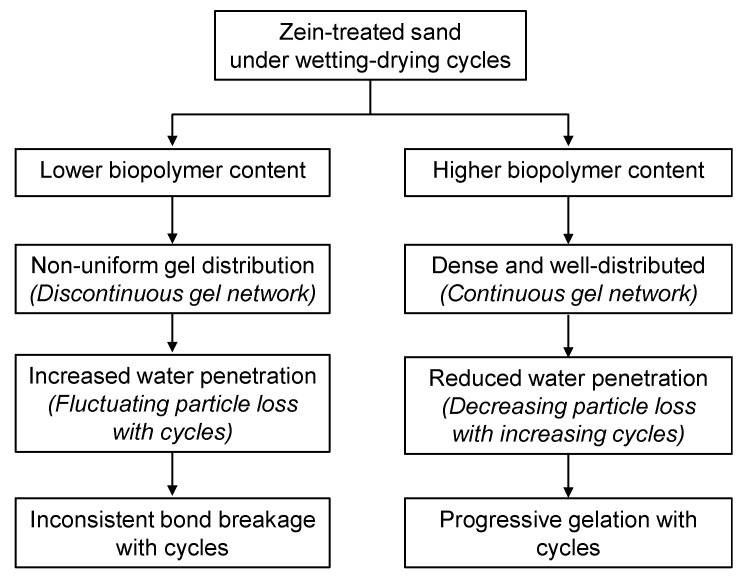
Schematic representation of the proposed durability mechanism of zein-treated sand under wetting–drying cycles.

The durability mechanism of zein-treated sand is illustrated in Figure 12. A lower biopolymer–sand ratio at 1% biopolymer content can produce a non-uniform and discontinuous gel coating around sand particles, resulting in weak and partially unbonded particles. Under repeated wetting–drying cycles, water penetrates along these unbonded pathways, resulting in fines detachment and progressive breakage of interparticle bonds. Consequently, non-uniform bonding leads to an inconsistent and generally low durability index as the number of cycles increases. In contrast, a higher biopolymer–sand ratio at 3% biopolymer content forms a dense and continuous gel network that uniformly coats and bridges sand particles. Consequently, the durability index initially increases and then remains relatively stable with additional cycles, indicating progressive strengthening. Nonetheless, repeated wetting retards biopolymer gelation and weakens interparticle bonding because of continued water penetration through the pore spaces.

## 5. Conclusions

This study investigated the strength and degradation characteristics of sand treated with different biopolymer binders. XG- and zein-treated sand specimens were subjected to repeated wetting and drying cycles. The mass and dimensions of the specimens were monitored to examine their volumetric variation across different numbers of wetting and drying cycles. The strength and degradation characteristics of the treated specimens were examined based on the number of cycles, curing period, biopolymer type, and biopolymer content.

The results showed that the bulk unit weight of the zein-treated sand remained almost constant in the drying phase and varied during the wetting phase. The XG-treated sand showed early strength improvement regardless of the biopolymer content. The zein-treated sand demonstrated gradual gelation, achieving higher compressive strength than the XG-treated specimens after 28 days of curing. The XG-treated sand rapidly deformed after the first wetting phase due to its strong affinity for water. The compressive strength of the zein-treated sand under wetting–drying conditions increased with the number of cycles. The compressive strength of the cycled specimens was lower than that of the uncycled specimens due to delayed gelation and the formation of a protein network. Similarly, the ductility index of the cycled specimens decreased with increasing biopolymer content. Zein-treated sand prepared with 3% biopolymer content showed a higher durability index after 10 cycles than that prepared with 1% biopolymer content, due to enhanced biopolymer–particle bonding. Therefore, increasing zein content can improve the strength and durability of sand subjected to moisture fluctuations.

## Figures and Tables

**Table 1:** Physical properties of biopolymers.

Properties	Xanthan Gum	Zein
pH	6.0–8.0	≥11.5
Melting point [°C]	64.43	266–283
Molecular weight [kDa]	2000–5000	20–24
Density [g/cm^3^]	1.5	1.23
Solubility in 25 °C pure water [mg/L]	10,000	~0.1

The solubility data were taken from Liu et al. [30] and Sworn [31].

**Table 2:** Dry unit weights of biopolymer-treated specimens.

Biopolymers	Unit Weight [kN/m^3^]	
	BC = 1%	BC = 3%
Xanthan gum	16.8	15.7
Zein	18.4	17.7

BC denotes the biopolymer content.

## Data Availability

The original contributions presented in this study are included in the article. Further inquiries can be directed to the corresponding author.
